# Serendipitous Discovery: A Case of Twin Congenital Pseudarthrosis of the Clavicle in Saudi Arabia and Literature Review

**DOI:** 10.7759/cureus.56641

**Published:** 2024-03-21

**Authors:** Ammar Abed Alqader Okasha, Areeg Kamal Abbas, Mohamad Alsamal, Idris Sula

**Affiliations:** 1 Internal Medicine, Dr. Sulaiman Al Habib Medical Group, Buraydah, SAU; 2 Basic Sciences, Sulaiman Al Rajhi University, Al Bukayriyah, SAU

**Keywords:** congenital pseudarthrosis of the clavicle, congenital, newborn, congenital clavicle abnormality, collarbone pseudarthrosis

## Abstract

Congenital pseudarthrosis of the clavicle (CPC) is a rare disorder with an unknown etiology, which is caused by a failure of the clavicle ossification nuclei union process. This is the first CPC twin instance documented in Saudi Arabia, and the fourth overall. In the 33rd week, a set of twins was born prematurely with respiratory distress syndrome. They were both admitted to the neonatal intensive care unit. X-rays of the chest were taken, and the clavicular deformity was discovered. Because the twins were born via a cesarean section, a traumatic clavicular fracture was ruled out. The family members were evaluated, and the same defect was discovered in the father as well, highlighting a genetic predisposition.

## Introduction

Congenital pseudarthrosis of the clavicle (CPC) is a rare disorder of unknown etiology, which is commonly diagnosed in the early years of life [1-3]. CPC is triggered by the failure of the union process of the ossification nuclei of the clavicle [4,5]. The first ever documented case was in 1910 by Fitzwilliams, and currently, CPC stands as an exceptionally uncommon congenital shoulder girdle deformity, with around 323 global cases documented [4,6,7]. Initially, there was an assumption that CPC was more frequent in females compared to males [3,7]. Nevertheless, subsequent observations indicated an equal ratio of males to females at 1:1 [5]. On the other hand, it predominantly affects the right side for unknown reasons [6]. Only three CPC cases have been reported so far in Saudi Arabia and were diagnosed during childhood [6,8,9]. Patients usually present early in childhood with a painless swelling over the middle third of the clavicle, which tends to increase in size as the child grows [5,9]. Differential diagnoses of CPC include obstetric fracture (which tends to rapidly heal with an exuberant callus), cleidocranial dysostosis, neurofibromatosis, and post-traumatic nonunion [5,6,8]. The defining feature of CPC is the existence of a painless lump located above the right clavicle. In cases of CPC, the distance between the sternum and acromion is noticeably reduced. This swelling is thought to result from the enlargement of bone ends and the overlapping of these fragments [5,8]. There have been reports that the patients might be asymptomatic during their entire life and with painless and normal shoulder range of motion [2,5]. Around a quarter of patients experience additional symptoms like pain, reduced functionality, and restricted range of motion. These symptoms seem to slightly increase with age. When examining smaller sets of CPC cases, five instances of thoracic outlet syndrome (TOS) resulting from CPC were identified [5].

The occurrence of genetic, chromosomal pattern, or familial inheritance is not well defined, most likely sporadic (constituting less than 3% of cases), and the etiology and prevalence of CPC have not been established yet [5]. Up to our research, we have come across a well-documented family in which multiple members were afflicted by CPC as well as a case involving CPC occurring in a pair of twins [10]. Moreover, the first documented case of two siblings being diagnosed with CPC was reported in 2008; those siblings were asymptomatic and had normal mobility of the shoulder [3].

On average, caregivers (such as parents, obstetricians, and family doctors) observed the deformity for the first time when the affected individual was around 2.1 years old. The average age at which the case was brought to the orthopedic surgeon was approximately around 4.6 years. A considerable majority of symptomatic patients underwent surgical intervention for treatment, amounting to 92.5% of cases [5].

## Case presentation

This case report is about a set of twins who were born prematurely in March 2022 at 33 weeks and presented with respiratory distress syndrome (RDS) and suspected sepsis. The twins were delivered according to emergency lower segment cesarean section (LSCS) guidelines, undertaken on account of imminent labor in the context of a twin pregnancy. Initial resuscitative interventions were promptly executed in alignment with Neonatal Resuscitation Program (NRP) guidelines. Both twins were admitted to the neonatal intensive care unit (NICU).

First case

Twin number 1 was a preterm male neonate delivered at 33 weeks and directly admitted to the NICU due to RDS with Apgar scores of 4 and 7 at one and five minutes, respectively. Clinical examination unveiled the following findings: the presence of a simian crease bilaterally across the neonate's palms and signs of respiratory compromise were evident such as labored breathing and grunting. The neonate was born with a weight of 2.23 kilograms, with a head circumference of 31 centimeters, and a length of 48 centimeters. A bilateral evaluation of the red reflex in ocular examination was normal. Neurological examination showed a flat and open fontanelle, with reactive pupillary reflexes. The neurological evaluation overall corroborated intact neurological function, satisfactory muscle tone, and reflex responses.

**Figure 1:**
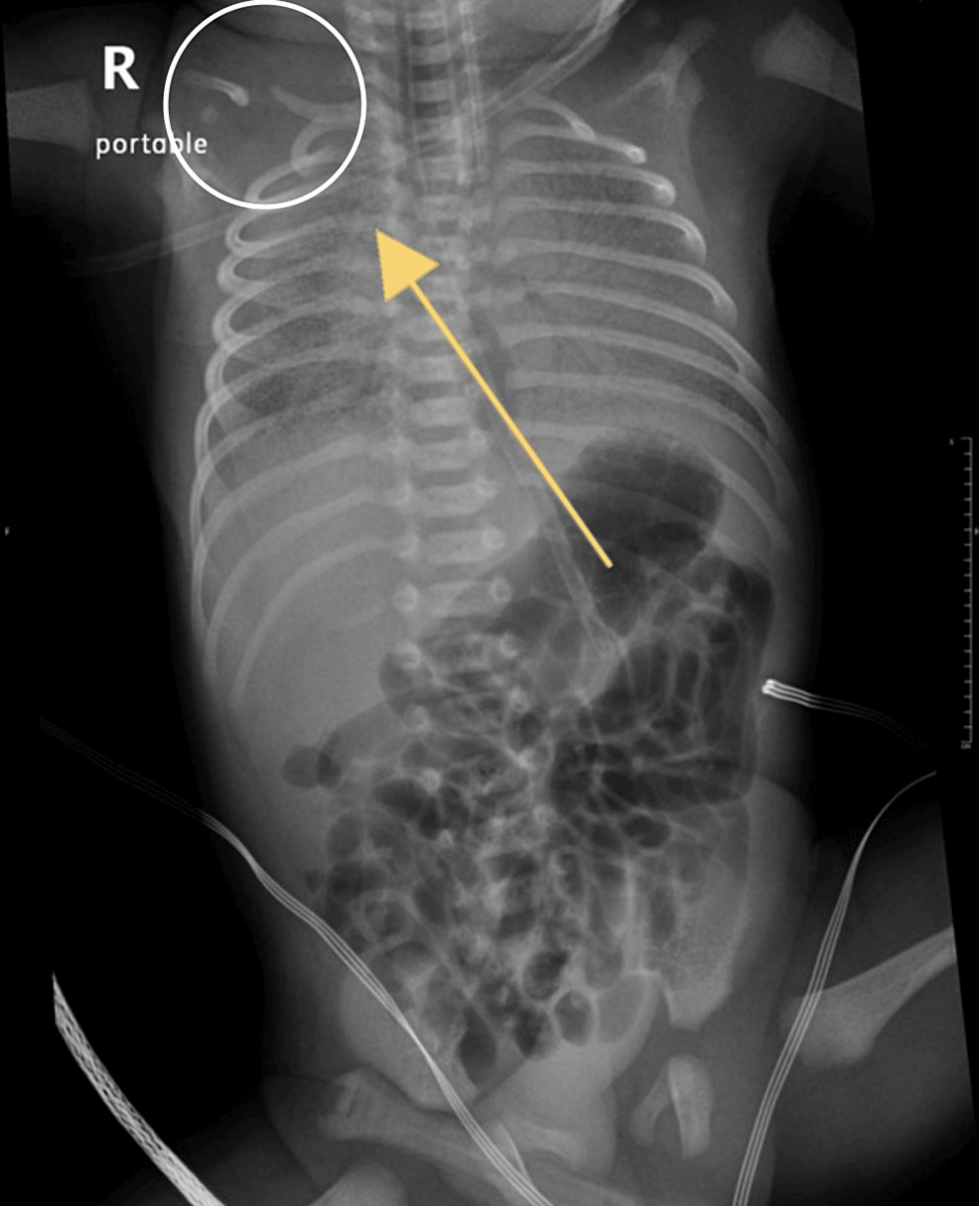
Chest X-ray of the first case showing the deformity of the clavicle on the right side.

In the thoracic domain, clinical scrutiny identified respiratory distress represented by tachypnea and suprasternal and subcostal retractions, indicative of increased respiratory effort. Notably, bilateral auscultation revealed diminished air entry coupled with desaturation. The cardiac assessment demonstrated a capillary refill time of three seconds, palpable femoral pulses, and a heart rate of 164 beats per minute. Normal first and second heart sounds were auscultated. Abdominal examination yielded findings consistent with lax musculature, soft texture, and absence of organomegaly. Notably, incidental finding of CPC was noted on the chest X-ray (Figure 1).

Second case

Twin number 2 was a preterm male neonate delivered at 33 weeks and directly admitted to the NICU due to RDS with Apgar scores of 8 and 9 at one and five minutes, respectively. As desaturation and grunting were noticed, the baby was given continuous positive airway pressure (CPAP) at a positive end-expiratory pressure (PEEP) of 6 cm H2O. The birth weight was 1.96 kilograms, with a head circumference of 31 millimeters, and a length of 45 centimeters. The red reflex was normal bilaterally. A neurological examination indicated an open and flat anterior fontanelle, with pupils responding to light stimuli bilaterally. A complete neurological evaluation confirmed normal brain function, as well as normal muscle tone and reflex responses.

**Figure 2:**
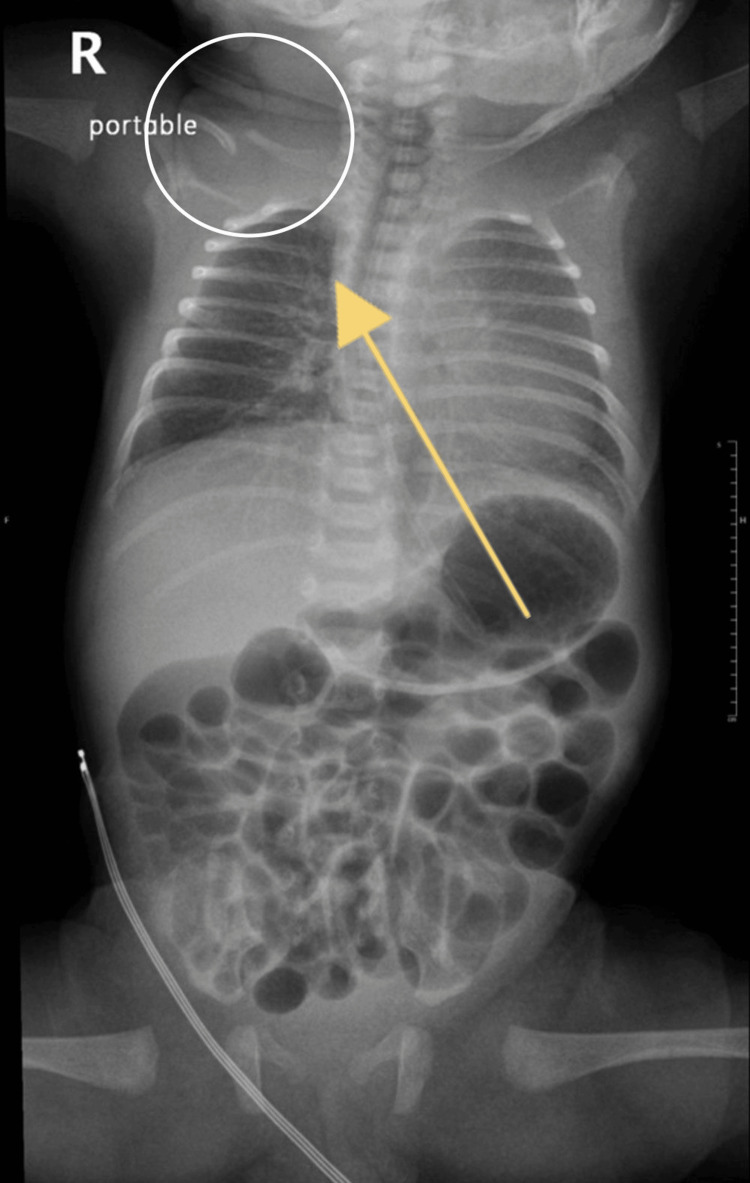
Chest X-ray of the second case showing the deformity of the clavicle on the right side.

In terms of respiratory assessment, the neonate exhibited features consistent with respiratory distress, typified by tachypnea and visible retractions of the suprasternal and subcostal regions. Auscultation of the chest detected diminished air entry bilaterally, concomitant with episodes of desaturation. Cardiac evaluation encompassed a capillary refill time of three seconds, discernible femoral pulses, and a heart rate of 164 beats per minute. Auscultatory findings indicated normal first and second heart sounds. Abdominal examination ascertained lax abdominal musculature, a supple texture, and the absence of organomegaly. Notably, incidental finding of CPC was noted on the chest X-ray (Figure 2).

Later on during the admission, a pediatric orthopedic referral was done for both twins with the following response: the patient has been diagnosed with congenital pseudarthrosis of the clavicle, with a low likelihood of a fracture-related etiology. To gain further insights, it is crucial to gather a comprehensive family history regarding the occurrence of fractures within the patient's lineage. This information can help determine whether the condition is exclusive to the clavicle or if other skeletal sites are affected, supporting the diagnosis of pseudarthrosis, a condition that often requires minimal intervention. It is advisable to schedule a follow-up clavicle X-ray at the age of six months to monitor the condition's progression.

The twins were discharged after a nine-day hospital stay, with their overall health in good condition. Significantly, the patients' family history was noteworthy, revealing a positive familial predisposition to right clavicular pseudoarthrosis, with two siblings and the father having experienced CPC.

Consent was obtained from the parents for the publication of this case report.

## Discussion

As per sources, this is the first twin case of CPC reported in Saudi Arabia and the fourth in total. The diagnosis was made shortly after birth. The delivery was by cesarean section and the twins were premature and admitted shortly after to NICU as they suffered RDS. Chest X-rays were done and the clavicular defect was noticed. The most common differential diagnosis of CPC is acute clavicular fracture from birth trauma. The current case is non-traumatic as it was an uneventful cesarean section. The X-ray showed sclerotic closure of the medullary canal without forming reactional bone calluses and the clinical and radiological findings were typical of CPC. Moreover, both neonates had unilateral right-sided involvement, and in both cases, the right clavicle’s medial piece was above the lateral piece, which is typically found in CPC [4,5,8]. However, due to the very rare occurrence of CPC, it is unknown why the incidence of the right clavicle is much more common than the left clavicle. Furthermore, determining the precise prevalence is challenging due to its typically asymptomatic nature, lack of associated morbidity, and negligible impact on quality of life. It is worth mentioning that this case report highlights that at least 1% of reported CPC cases have a positive familial history. Furthermore, a clear genetic pattern has not yet been associated with CPC [8]. Undiagnosed CPC cases might result in severe complications, including TOS [8]. Moreover, the incidence of impaired function was as high as 22% [8]. Therefore, we recommend considering familial inheritance patterns in all presenting cases and conducting thorough investigations accordingly. Certainly, it is advisable to obtain a detailed family history and it should also be included as part of the screening process if any suspicion arises.

This case report lacks further follow-up of the twins.

## Conclusions

Further investigation by the pediatric orthopedist confirmed the diagnosis of CPC in both twins. Moreover, both neonates had unilateral right-sided involvement, highlighting the fact that CPC predominantly affects the right side. Furthermore, even though most of the cases are sporadic, in our case, the father was diagnosed with CPC as well, which highlights a genetic predisposition.
